# Aspirin vs. Low-Molecular-Weight Heparin in Orthopedic Trauma: A Systematic Review and Meta-Analysis

**DOI:** 10.3390/jcm15176768

**Published:** 2026-08-31

**Authors:** Mohammed Alenezi, Abdulrahman O. Al-Naseem, Abdulrahman Alenezi, Yahya Ali, Abdullah Almehandi, Anthony Albers, Owayed Al Mutairi, Ali Jarragh

**Affiliations:** 1 Department of General Surgery, Al-Adan Hospital, Kuwait City 52700, Kuwait; 2 Division of Orthopaedic Surgery, McGill University, Montreal, QC H3A 1B9, Canada; 3 Kuwait Institute of Medical Specialization, Kuwait City 13018, Kuwait; 4 Faculty of Life Science and Medicine, King’s College, London SE1 9RT, UK; 5 Department of Cardiovascular Science, University College London, London WC1E 6BT, UK; 6 Ministry of Health, Kuwait City 13001, Kuwait; 7 Department of Surgery, Health Sciences Centre, Faculty of Medicine, Kuwait University, Kuwait City 13001, Kuwait

**Keywords:** aspirin, LMWH, orthopedic trauma, venous thromboembolism, meta-analysis

## Abstract

**Introduction:** Venous thromboembolism (VTE) remains a major complication following orthopedic trauma and fracture surgery. Low-molecular-weight heparin (LMWH) is widely used for prophylaxis; however, aspirin (ASA) has emerged as a potential alternative due to its ease of administration and cost-effectiveness. This study aimed to compare the efficacy and safety of aspirin (ASA) versus low-molecular-weight heparin (LMWH) in orthopedic trauma patients. **Methods:** A systematic review and meta-analysis was conducted by searching PubMed, Cochrane Library, and Google Scholar. Randomized controlled trials and comparative observational studies evaluating aspirin (ASA) versus low-molecular-weight heparin (LMWH) for venous thromboembolism (VTE) prophylaxis in orthopedic trauma patients were included. Outcomes assessed included overall venous thromboembolism (VTE), deep-vein thrombosis (DVT), pulmonary embolism (PE), fatal PE, mortality, bleeding complications, wound site infection, and hematoma formation. Pooled odds ratios (ORs) with 95% confidence intervals (CIs) were calculated using a random-effects model. Heterogeneity was assessed using the I(2) statistic. **Results:** Five studies comprising a heterogeneous orthopedic trauma population were included. No statistically significant difference between aspirin (ASA) and low-molecular-weight heparin (LMWH) in the incidence of venous thromboembolism (VTE) was noted (OR 0.52, 95% CI 0.02–12.25, *p* = 0.68). No significant differences were observed in the incidence of DVT between aspirin (ASA) and low-molecular-weight heparin (LMWH) (OR 1.00, 95% CI 0.46–2.16, *p* = 1.00). Pulmonary embolism (PE) (OR 0.96, 95% CI 0.72–1.28, *p* = 0.80), fatal PE (OR 0.55, 95% CI 0.18–1.65, *p* = 0.28), and mortality (OR 1.02, 95% CI 0.68–1.54, *p* = 0.91) were comparable between the two groups. Similarly, no significant differences were observed in bleeding complications (OR 0.95, 95% CI 0.86–1.05, *p* = 0.33) or wound complications (OR 1.05, 95% CI 0.81–1.37, *p* = 0.70). Hematoma formation showed a trend favoring aspirin (ASA) (OR 0.36, 95% CI 0.13–1.02, *p* = 0.05), although this did not reach statistical significance. **Conclusions:** Aspirin (ASA) may be a reasonable alternative to low-molecular-weight heparin (LMWH) for venous thromboembolism (VTE) prophylaxis in selected orthopedic trauma patients, with comparable outcomes for pulmonary embolism (PE), mortality, bleeding and wound complications. Larger high-quality randomized trials with standardized protocols in clearly defined orthopedic trauma populations are warranted to confirm these findings and guide clinical decision-making.

## 1. Introduction

Venous thromboembolism (VTE) remains a significant and potentially fatal complication following orthopedic trauma, contributing to increased morbidity, mortality, and healthcare burden. Although low-molecular-weight heparin (LMWH) has traditionally been recommended by clinical guidelines for thromboprophylaxis in trauma patients, the recent literature suggests that aspirin (ASA) may be similarly effective [1,2], with the advantages of oral administration, lower cost, favorable safety profile, and improved patient compliance. However, the optimal pharmacological prophylaxis for preventing venous thromboembolism (VTE) in this population remains debated, as recommendations vary across expert societies and clinical contexts [3]. Although recent high-quality trials have renewed interest in aspirin (ASA) for thromboprophylaxis after orthopedic trauma, uncertainty persists regarding its role across the broad spectrum of trauma patients, particularly those with varying injury severity, fracture patterns, and baseline thromboembolic risk. Furthermore, much of the existing evidence has been derived from individual randomized trials or elective arthroplasty populations, limiting its generalizability to heterogeneous trauma cohorts encountered in routine clinical practice [4]. Randomized trials and meta-analyses indicate that aspirin (ASA) and low-molecular-weight heparin (LMWH) have comparable efficacy in preventing venous thromboembolism (VTE) following orthopedic trauma, specifically deep-vein thrombosis (DVT) and pulmonary embolism (PE), without significant differences in overall mortality or bleeding complications [5]. Despite this growing body of literature, there is no comprehensive synthesis integrating both randomized and real-world evidence in trauma patients.

Therefore, we performed a systematic review and meta-analysis to compare the safety and efficacy of aspirin (ASA) versus low-molecular-weight heparin (LMWH) for thromboprophylaxis in orthopedic trauma.

## 2. Methods

This systematic review and meta-analysis were conducted in accordance with the Preferred Reporting Items for Systematic Reviews and Meta-Analyses (PRISMA) guidelines [6]. This protocol is registered in PROSPERO (International Prospective Register of Systematic Reviews; CRD420261432809). PRISMA checklist is provided in Supplementary Table S1.

### 2.1. Eligibility Criteria

Randomized controlled trials and observational studies comparing aspirin (ASA) with low-molecular-weight heparin (LMWH) in patients with orthopedic trauma and reporting relevant outcomes were included. Exclusions: non-English studies, patients undergoing arthroplasty or elective orthopedic procedures, lack of direct comparison, studies with overlapping populations, and studies not reporting relevant outcomes of interest. Additionally, reference lists of all included studies were examined to identify further relevant articles. Duplicate records were removed, and two independent authors screened each eligible study for final inclusion.

### 2.2. Literature Search Strategy and Selection of Studies

Two independent authors conducted a database search in PubMed, Google Scholar, and CENTRAL, with the final search completed on 5 April 2026. The ICTRP and ISRCTN were screened for unpublished trials. No language restrictions were applied. Search strategy included (*orthopedic trauma* OR *hip fracture* OR *pelvic fracture* OR *extremity fracture* OR *femoral neck fracture* OR *trochanteric fracture*) AND (*venous thromboembolism* OR *VTE* OR *deep vein thrombosis* OR *DVT* OR *pulmonary embolism* OR *PE*) AND (*low-molecular-weight heparin* OR *LMWH* OR *enoxaparin*) AND (*aspirin* OR *ASA* OR acetylsalicylic acid*). Titles and abstracts of all identified studies were independently screened by two authors. Full-text articles of potentially relevant studies were obtained and evaluated against eligibility criteria. Discrepancies were resolved by consensus.

### 2.3. Data Extraction and Management

Data extraction followed the Cochrane data collection form for intervention reviews. A structured spreadsheet was developed, pilot-tested, and refined using randomly selected articles. The data extraction sheet included:

1. Study information: First author, publication year, corresponding author’s country, journal, study design, sample size, intervention type, and comparator.
2. Baseline demographics: Age, gender, fracture type, sample size, intervention, and follow-up duration.
3. Outcomes assessed: Overall venous thromboembolism (VTE), deep-vein thrombosis (DVT), pulmonary embolism (PE), fatal PE, mortality, bleeding complications, wound site infection, and hematoma formation.

### 2.4. Data Analysis and Assessment of Heterogeneity

Cochrane Review Manager 5.3 was used to perform the meta-analysis. Pooled odds ratios (ORs) with 95% confidence intervals (CIs) were calculated for all outcomes using a random-effects Mantel–Haenszel model. Heterogeneity was assessed using the I^2^ statistic and interpreted in accordance with the Cochrane Handbook: 0–40% might not be important, 30–60% may represent moderate heterogeneity, and 50–90% may represent substantial heterogeneity [7].

### 2.5. Quality Assessment and Sensitivity Analysis

Risk of bias was evaluated with ROB2 for randomized studies and the Newcastle–Ottawa scale for observational studies [8,9]. The certainty of evidence for each outcome was assessed using the GRADE approach. The certainty of evidence was downgraded when concerns were identified regarding risk of bias, inconsistency, indirectness, imprecision, and publication bias. Certainty was categorized as high, moderate, low, or very low. Two independent authors conducted the quality assessments, resolving disagreements by consensus.

Leave-one-out sensitivity analyses were conducted to evaluate the influence of individual studies on the pooled effect estimates and heterogeneity. Each study was sequentially excluded and the meta-analysis was repeated to assess the robustness of the findings.

## 3. Results

### 3.1. Screening of the Included Studies

**Figure 1:**
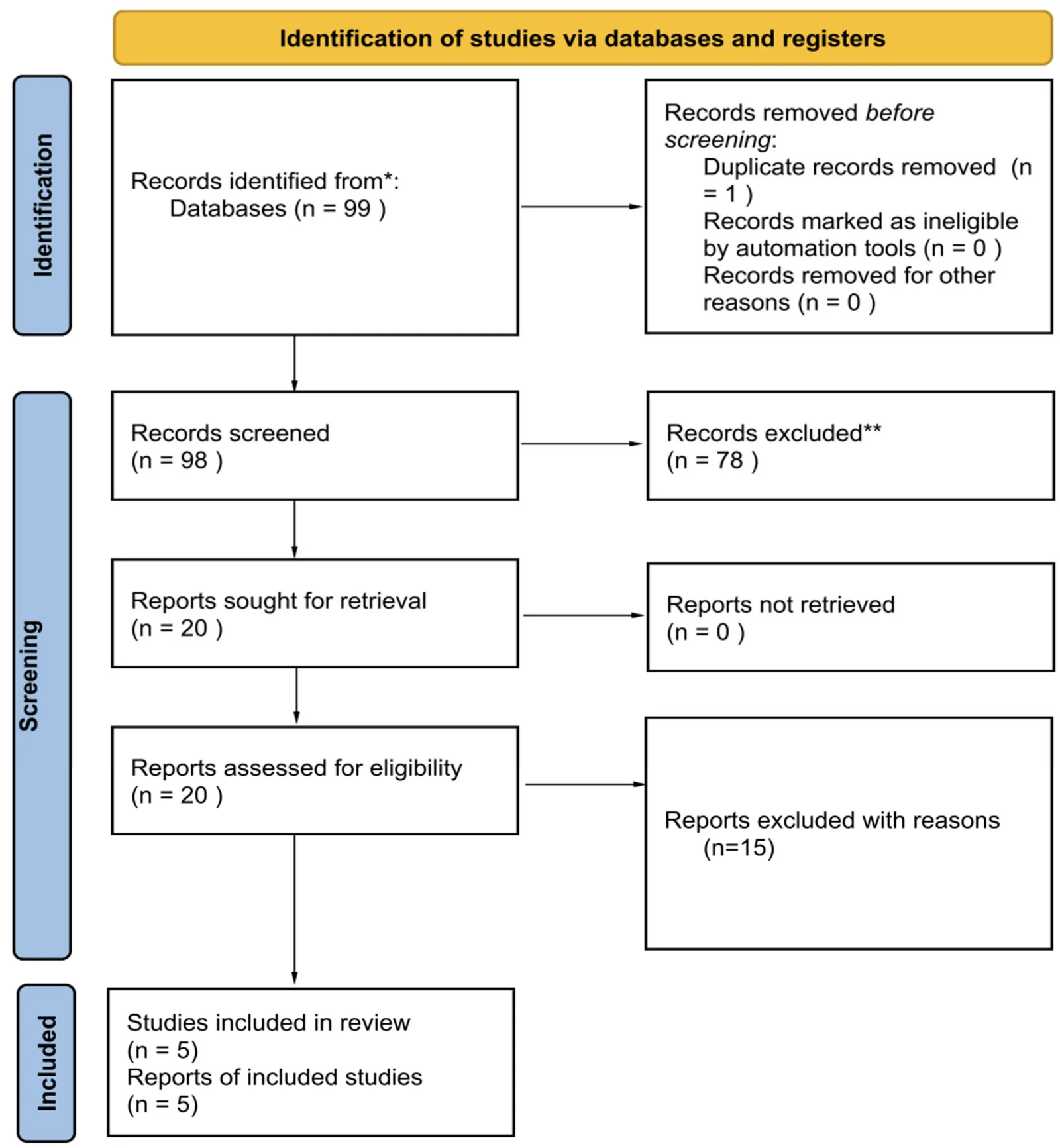
PRISMA flow chart of the included studies. * = Records identified, ** = Records excluded.

The comprehensive search of PubMed, the Cochrane library, and Google Scholar yielded 99 records. After excluding duplicates, 98 records were screened based on titles and abstracts, resulting in the exclusion of 78 articles. Subsequently, 20 full-text articles were assessed for eligibility, after which 15 articles were excluded based on pre-specified criteria. Finally, five studies were included in the systematic review after the inclusion criteria were met (Figure 1).

### 3.2. Characteristics of the Included Studies

Five articles investigating aspirin (ASA) vs. low-molecular-weight heparin (LMWH) were included in our study. The sample sizes ranged from 144 to 12,211 patients. The included studies comprised randomized controlled trials (RCTs), retrospective cohort studies, and prospective cohort studies. Follow-up durations ranged from 35 days to 90 days post-injury or surgery. Outcomes assessed included venous thromboembolism (VTE), bleeding complications, mortality, and wound-related complications, providing a comprehensive overview of the safety and efficacy of anticoagulant strategies in diverse orthopedic injury populations. The summary characteristics of the included studies are reported in Table 1. 

### 3.3. Outcomes

#### 3.3.1. VTE

**Figure 2:**
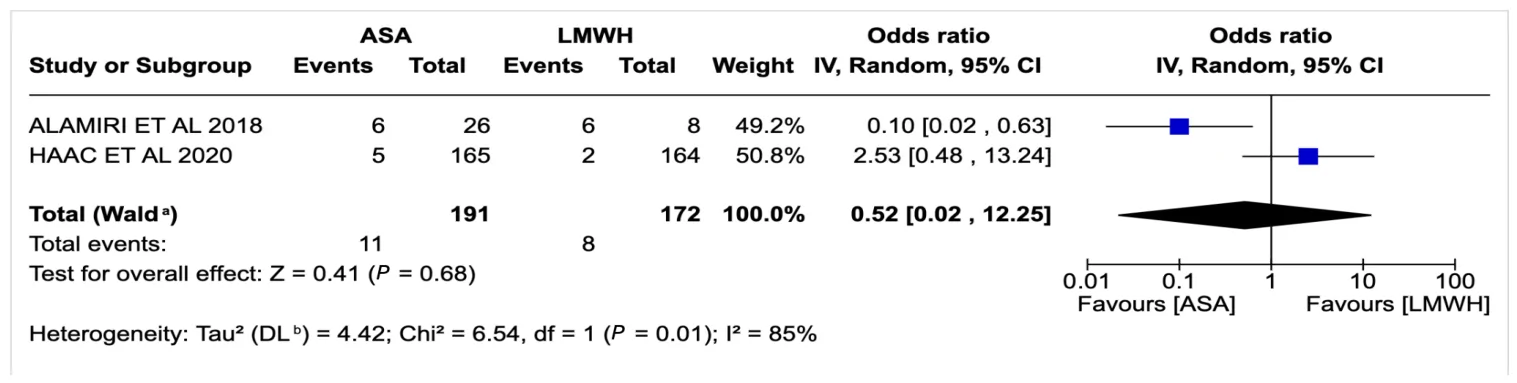
Forest plot of odds ratio of VTE outcome [1,13]. a = Wald method, b = DerSimonian–Laird estimator.

The pooled odds ratio of venous thromboembolism (VTE) was 0.52 with a 95% confidence interval (CI) ranging from 0.02 to 12.25 (Figure 2). The result was not statistically significant (*p* = 0.68). Substantial heterogeneity was observed (I^2^ = 85%, *p* = 0.01).

#### 3.3.2. All-Cause Mortality

**Figure 3:**
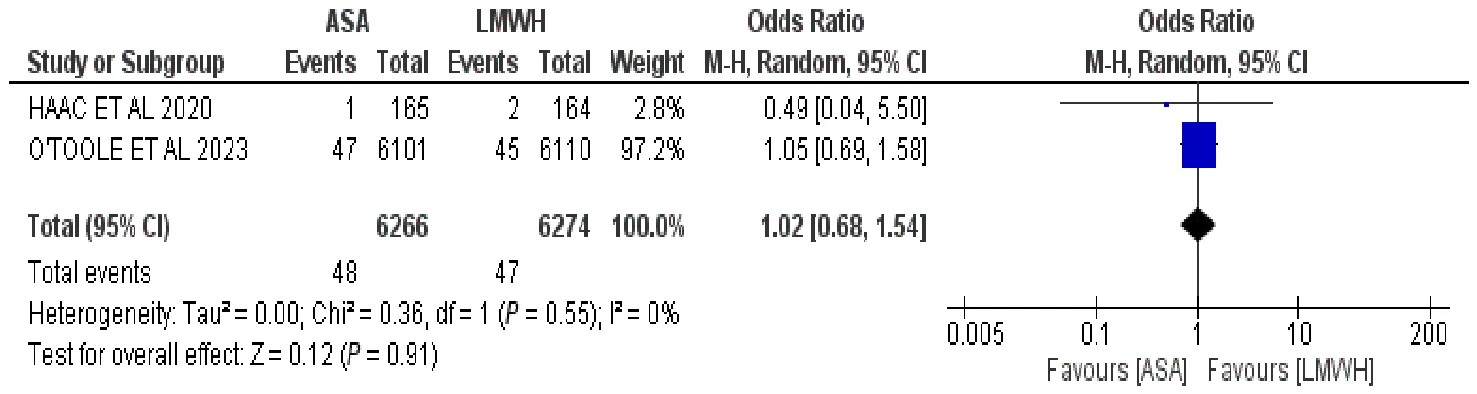
Forest plot of the odds ratio of all-cause mortality outcome [1,10].

The pooled odds ratio of all-cause mortality was 1.02 with a 95% confidence interval (CI) ranging from 0.68 to 1.54 (Figure 3). The result was not statistically significant (*p* = 0.91). No statistical heterogeneity was observed (I^2^ = 0%, *p* = 0.55).

#### 3.3.3. Bleeding Complications

**Figure 4:**
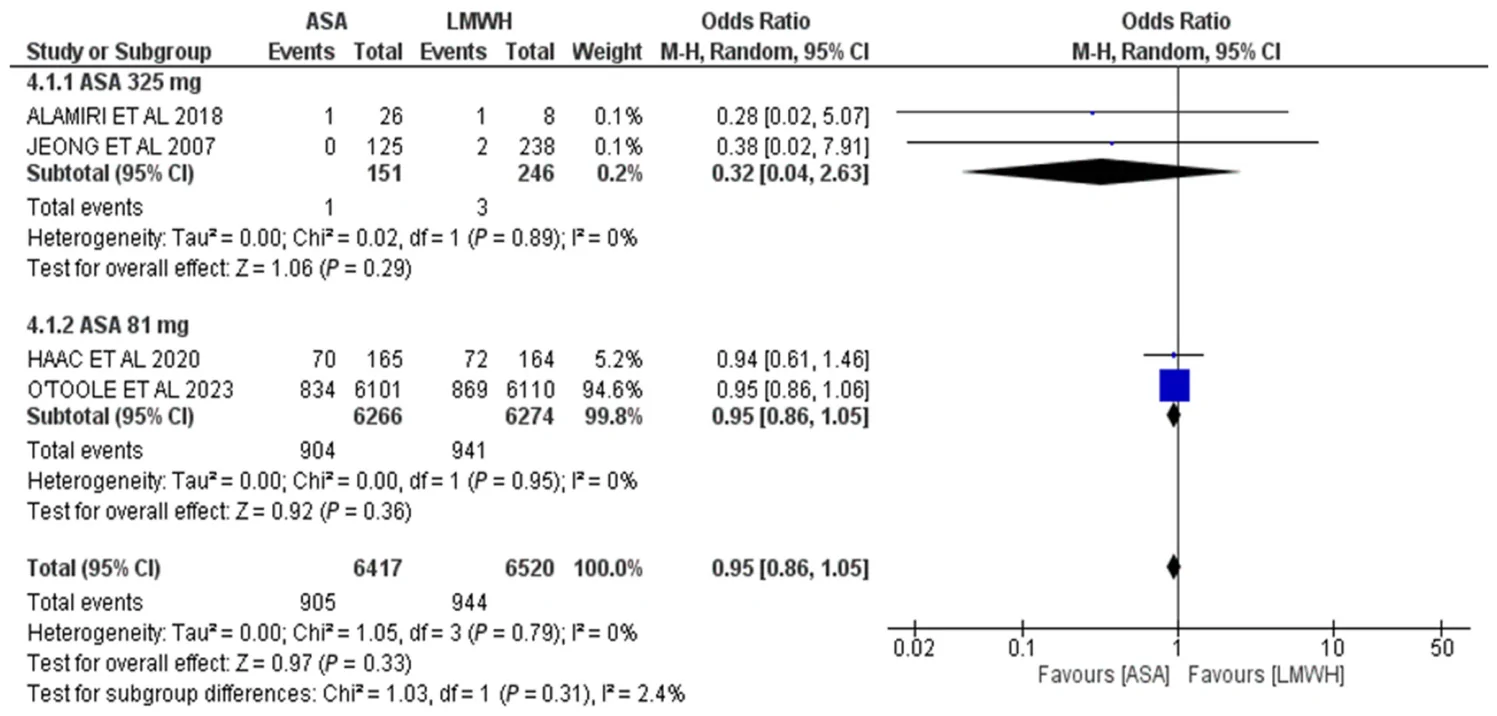
Forest plot and subgroup plots of the odds ratio of bleeding complication outcome [1,10,12,13].

The pooled odds ratio for bleeding complications was 0.95 with a 95% confidence interval (CI) ranging from 0.86 to 1.05 (Figure 4). The result was not statistically significant (*p* = 0.33). No statistical heterogeneity was observed (I^2^ = 0%, *p* = 0.79). An aspirin-dose subgroup analysis was performed for bleeding complications.

#### 3.3.4. Wound Complications

**Figure 5:**
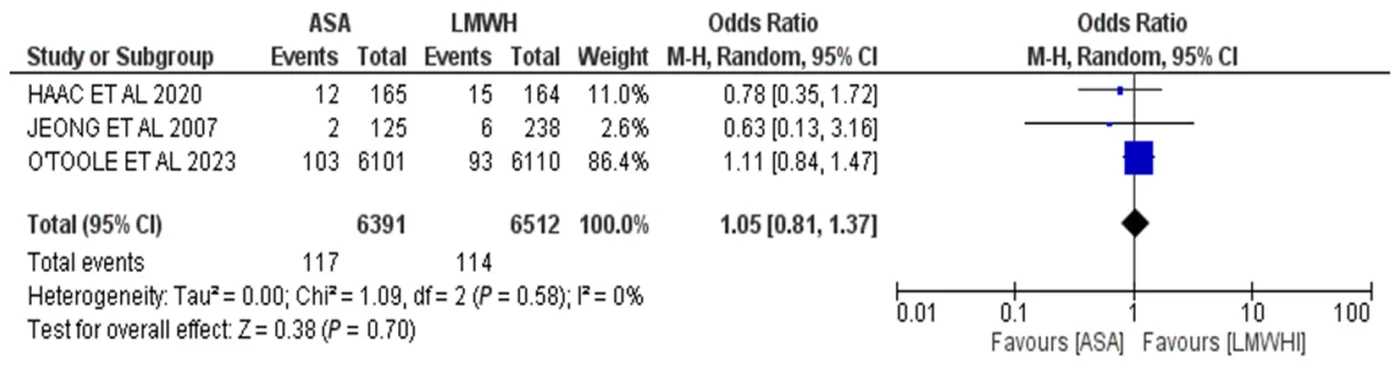
Forest plot of the odds ratio of wound complication outcome [1,10,12].

The pooled odds ratio of wound complications was 1.05 with a 95% confidence interval (CI) ranging from 0.81 to 1.37 (Figure 5). The result was not statistically significant (*p* = 0.70). No statistical heterogeneity was observed (I^2^ = 0%, *p* = 0.58).

#### 3.3.5. Hematoma Complications

**Figure 6:**
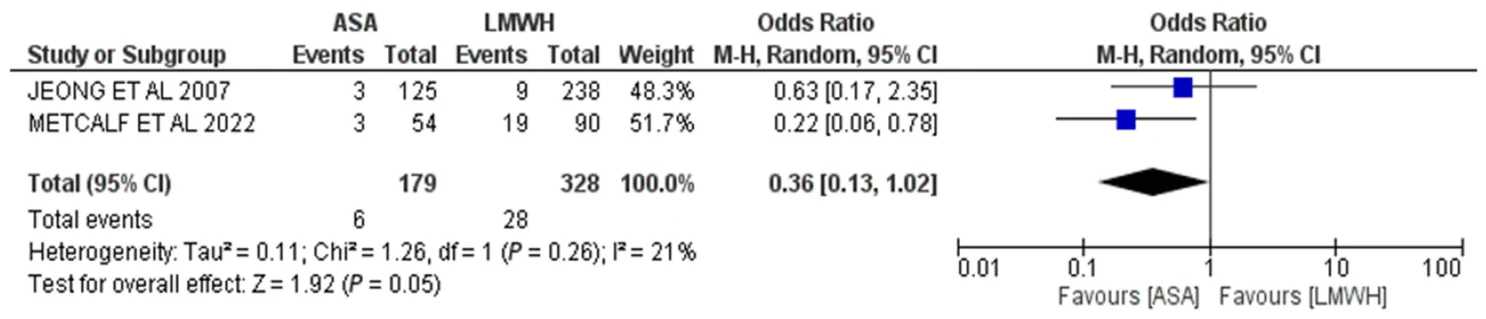
Forest plot of the odds ratio of the hematoma outcome [11,12].

The pooled odds ratio of hematoma was 0.36 with a 95% confidence interval (CI) ranging from 0.13 to 1.02 (Figure 6). The result was not statistically significant (*p* = 0.05). Low heterogeneity was observed (I^2^ = 21%, *p* = 0.26).

#### 3.3.6. PE

**Figure 7:**
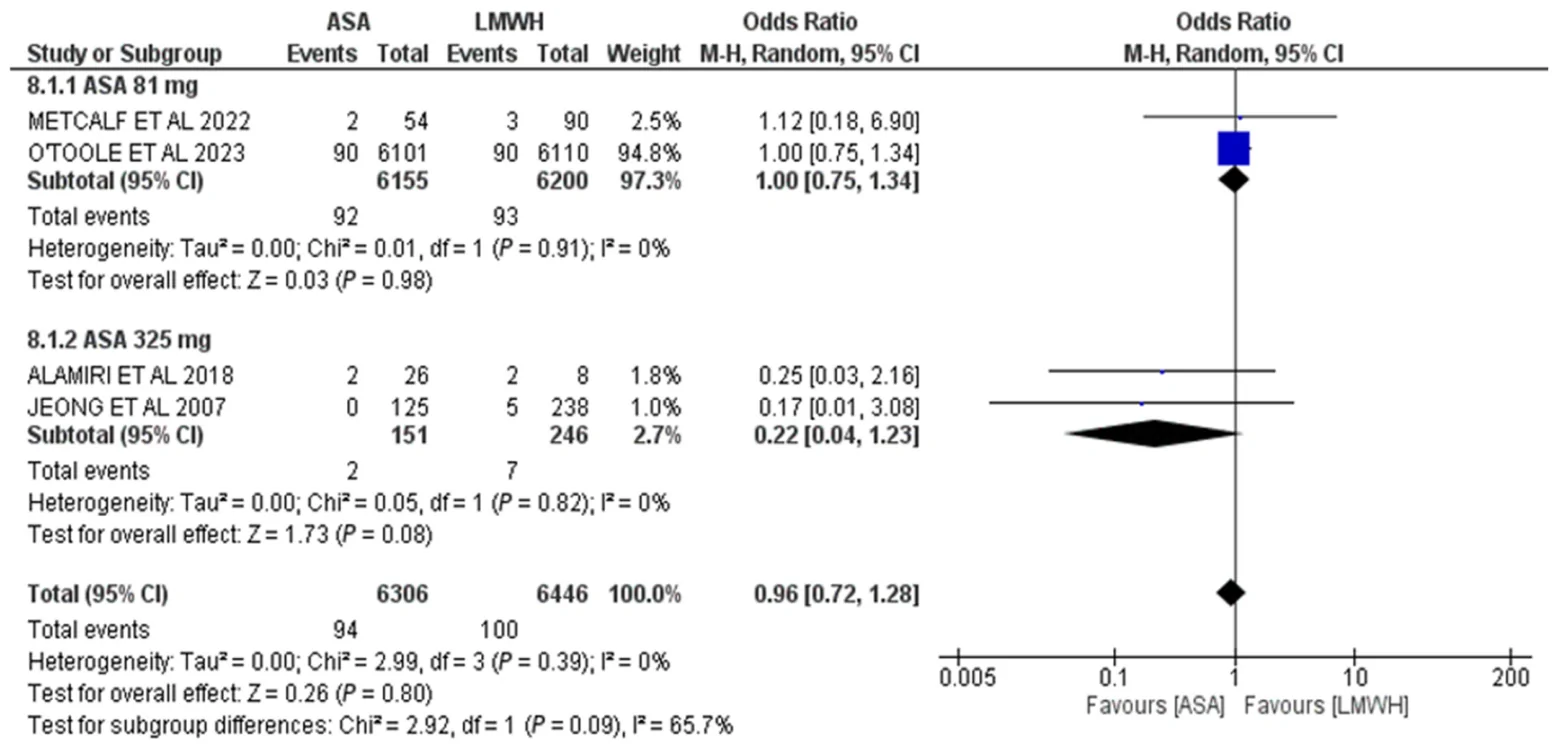
Forest plot and subgroup plots of the odds ratio of the PE outcome [10,11,12,13].

The pooled odds ratio of pulmonary embolism (PE) was 0.96 with a 95% confidence interval (CI) ranging from 0.72 to 1.28 (Figure 7). The result was not statistically significant (*p* = 0.80). No statistical heterogeneity was observed (I^2^ = 0%, *p* = 0.39). An aspirin-dose subgroup analysis was performed for PE.

#### 3.3.7. Fatal PE

**Figure 8:**
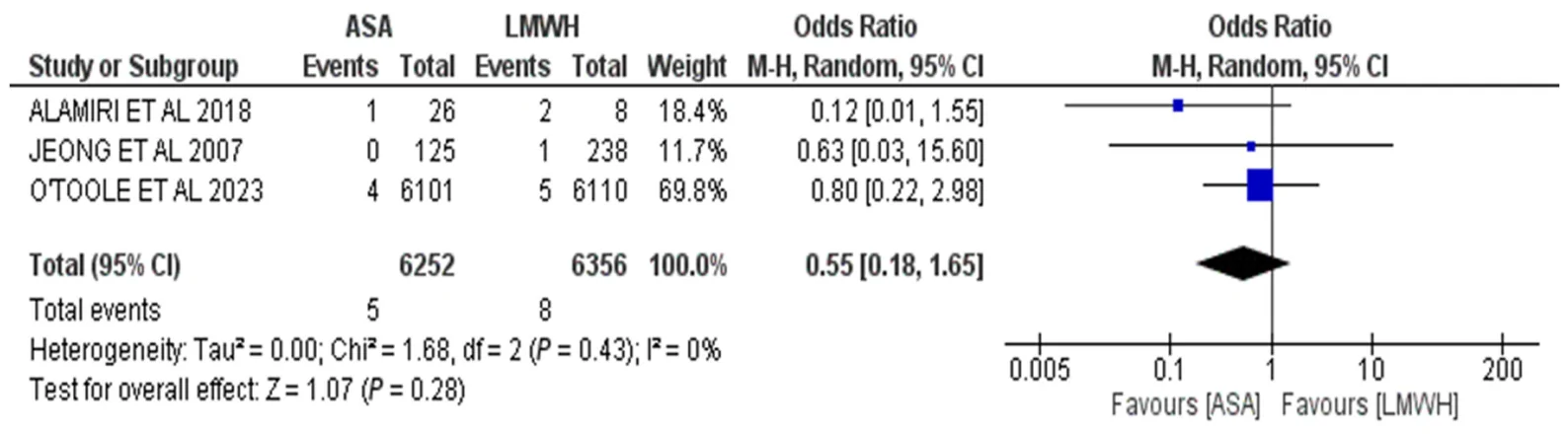
Forest plot of the odds ratio of the fatal PE outcome [10,12,13].

The pooled odds ratio of fatal PE was 0.55 with a 95% confidence interval (CI) ranging from 0.18 to 1.65 (Figure 8). The result was not statistically significant (*p* = 0.28). No statistical heterogeneity was observed (I^2^ = 0%, *p* = 0.43).

#### 3.3.8. DVT

**Figure 9:**
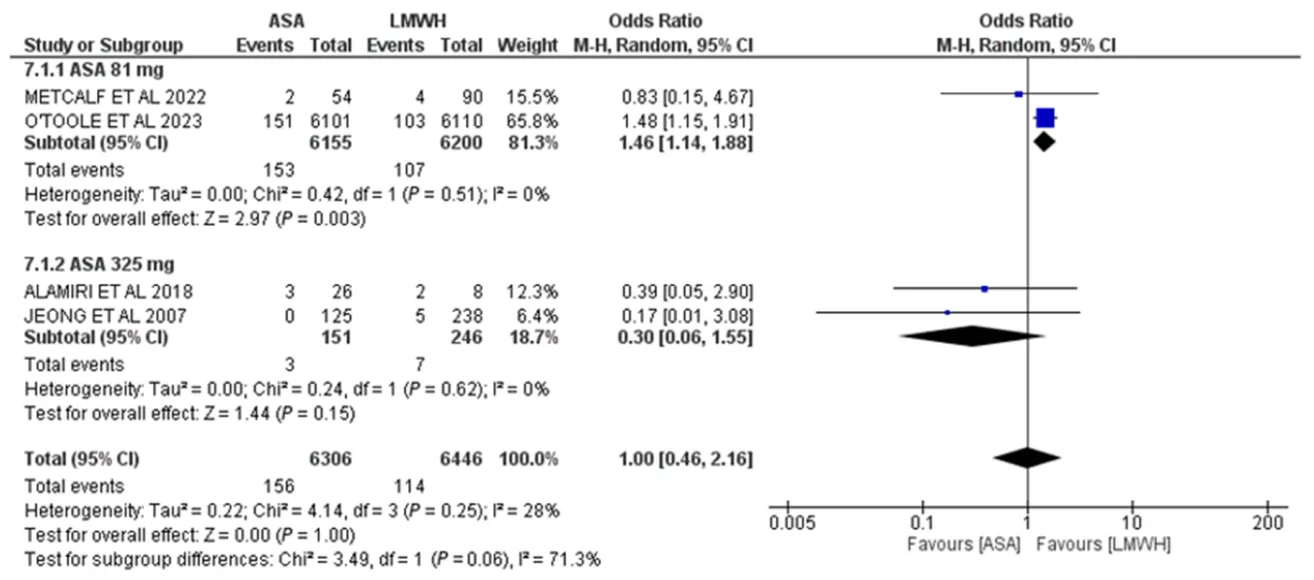
Forest plot and subgroup plot of the odds ratio of the DVT outcome [10,11,12,13].

The pooled odds ratio of deep-vein thrombosis (DVT) was 1.00 with a 95% confidence interval (CI) ranging from 0.46 to 2.16 (Figure 9). The result was not statistically significant (*p* = 1.00). Low heterogeneity was observed (I^2^ = 28%, *p* = 0.25). In leave-one-out sensitivity analysis, exclusion of Jeong et al. 2007 reduced heterogeneity to I^2^ = 3% [12]. An aspirin-dose subgroup analysis was performed for DVT.

### 3.4. Risk of Bias

**Figure 10:**
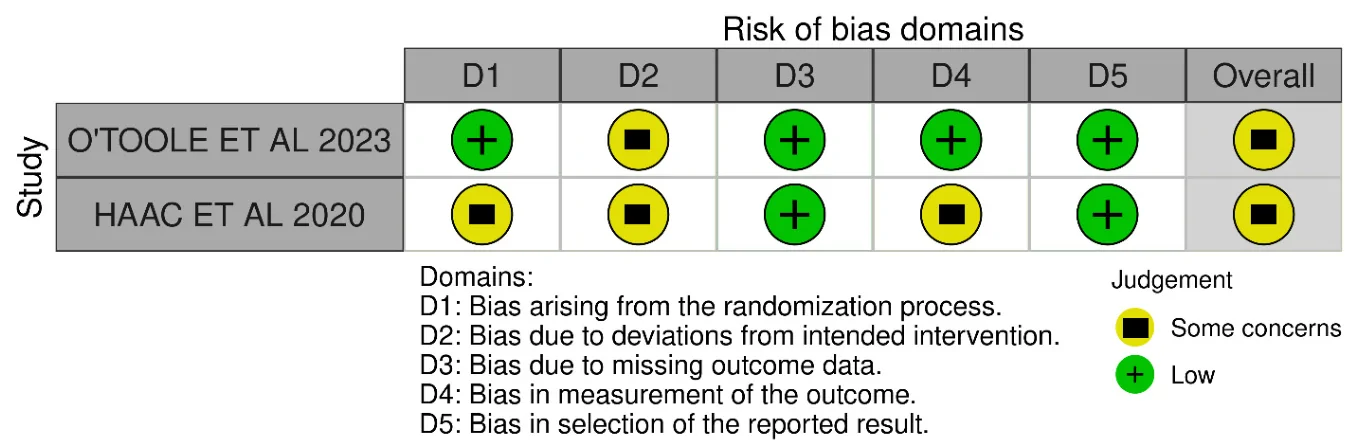
Risk of bias using RoB 2 [1,10].

The risk-of-bias assessment indicated that the observational studies were generally of high methodological quality according to the Newcastle–Ottawa Scale, scoring 7 to 8 out of 9 (Table 2), while both included RCTs were judged to have some concerns using RoB 2 (Figure 10). According to the GRADE assessment, the certainty of evidence ranged from very low to moderate (Table 3 and Table 4).

## 4. Discussion

In this meta-analysis of five comparative studies evaluating aspirin (ASA) versus low-molecular-weight heparin (LMWH) for venous thromboembolism prophylaxis in patients with orthopedic trauma, we found no statistically significant differences between the two agents in overall venous thromboembolism (VTE), mortality, bleeding complications, wound complications, or hematoma formation.

Our venous thromboembolism (VTE) analysis showed no significant overall difference between aspirin (ASA) and low-molecular-weight heparin (LMWH), but this finding should be interpreted more cautiously because heterogeneity was high. These findings align with a previous randomized trial, the PREVENT CLOT trial by O’Toole et al. [10], which reported similar deep-vein thrombosis (DVT) and pulmonary embolism (PE) rates; however, deep-vein thrombosis (DVT) occurred more often with aspirin (ASA), particularly distal deep-vein thrombosis (DVT). A recent meta-analysis by Haibier et al. reported low rates of deep-vein thrombosis (DVT) with the enoxaparin group post-sensitivity analysis [4]. Similarly, our findings are consistent with the ADAPT trial by Haac et al. [1], which did not demonstrate clear superiority of low-molecular-weight heparin (LMWH) over aspirin (ASA) using a patient-centered composite outcome, reinforcing the concept that aspirin may be a reasonable thromboprophylactic strategy after orthopedic trauma. Although the trial was smaller than the PREVENT CLOT trial and had less statistical power to detect differences in thromboembolic outcomes, it contributed to the growing evidence suggesting similar clinical outcomes with aspirin (ASA) and low-molecular-weight heparin (LMWH) in trauma populations.

The most clinically important finding in our analysis is the absence of a statistically significant difference in mortality between the two groups. The pooled estimate showed no significant difference in all-cause mortality. This result is supported by the largest and most influential trauma trial, the PREVENT CLOT trial, which showed that aspirin (ASA) was non-inferior to low-molecular-weight heparin (LMWH) for prevention of death at 90 days after operatively treated extremity fractures or pelvic/acetabular fractures. Additionally, Haibier et al. conducted a meta-analysis evaluating aspirin (ASA) versus low-molecular-weight heparin (LMWH) in hip and knee arthroplasty and hip fracture cohorts that showed no significant differences in 90-day all-cause mortality between the two agents [4]. From a clinical perspective, mortality is critical, and the absence of a significant difference between the two agents supports the growing acceptance of aspirin as a viable thromboprophylactic agent in appropriately selected trauma patients.

Our meta-analysis found no significant differences between aspirin (ASA) and low-molecular-weight heparin (LMWH) in bleeding complications, wound complications, or hematoma formation. Although the hematoma outcome showed a borderline signal favoring aspirin, this did not reach statistical significance. The PREVENT CLOT trial similarly found no significant difference in bleeding outcomes or wound complications between aspirin (ASA) and low-molecular-weight heparin (LMWH) [10]. The comparable efficacy of aspirin (ASA) and enoxaparin in preventing pulmonary embolism (PE) and major bleeding, together with aspirin’s superior safety profile regarding minor bleeding, aligns with the recommendations of guidelines such as those from the American Academy of Orthopaedic Surgeons (AAOS) and the American Society of Hematology (ASH), which suggest aspirin (ASA) as an option for patients undergoing major orthopedic surgery, particularly those at standard or low risk for venous thromboembolism (VTE), while low-molecular-weight heparin (LMWH) or Unfractionated heparin (UFH) favored in higher-risk major trauma patients [14,15]. Importantly, two recent secondary analyses of the PREVENT CLOT trial compared aspirin (ASA) and low-molecular-weight heparin (LMWH) across 11 high-risk and fracture-location subpopulations and among patients stratified by venous thromboembolism (VTE) risk using the Caprini score; neither analysis found a significant difference in mortality, deep-vein thrombosis (DVT), or pulmonary embolism (PE) rates [2,16], aspirin (ASA) was found to be more protective than low-molecular-weight heparin (LMWH) for all-cause mortality in patients with head or spine injuries, while low-molecular-weight heparin (LMWH) reduced the incidence of distal deep-vein thrombosis (DVT) in patients with severe head injuries, thoracic injuries and multiple injured patients with multiple injuries and an Injury Severity Score (ISS) > 16, but this did not remain significant after adjustment for multiple comparisons and were not associated with increased pulmonary embolism (PE) or mortality. This broad consistency across various patient subgroups is important given the ongoing debate about the optimal thromboprophylactic strategy in high-risk orthopedic patients and provides additional support for the potential use of aspirin (ASA) as an alternative to low-molecular-weight heparin (LMWH) for venous thromboembolism (VTE) prophylaxis in selected orthopedic trauma settings [17,18,19]. Systematic reviews further indicate that compared with Rivaroxaban and Warfarin, aspirin (ASA) does not significantly differ in terms of major bleeding, all-cause mortality, and wound-related events, and may even reduce minor bleeding and total bleeding; however, a subgroup analysis showed similar outcomes to Rivaroxaban and inferior outcomes compared with Warfarin [20]. Additionally, in total hip and knee arthroplasty, studies have demonstrated aspirin (ASA)’s non-inferiority to Rivaroxaban in venous thromboembolism (VTE) prevention with comparable bleeding rates [21]; however, it is important to note that both groups received Rivaroxaban for 5 days prior to randomization.

Collectively, our findings align closely with the existing orthopedic trauma literature. Aspirin (ASA) appears to offer comparable effectiveness to low-molecular-weight heparin (LMWH) in preventing clinically meaningful outcomes such as mortality and major thromboembolism, with a similar safety profile across bleeding and wound-related complications. Differences, where present, are subtle and likely driven by specific venous thromboembolism (VTE) subtypes rather than overall thrombotic risk [4,10].

This meta-analysis has several limitations that should be considered when interpreting the results: first, the inclusion of observational studies and the limited number of available studies, many of which had small sample sizes. Second, heterogeneous trauma populations with variability in injury patterns, follow-up durations, management strategies, and thromboprophylactic protocols. Patients with pelvic and acetabular fractures, lower extremity fractures, polytrauma, and isolated fractures were analyzed collectively despite having different baseline risks for venous thromboembolism (VTE) and bleeding. These variations in injury characteristics, severity, and patient risk profiles may have contributed to the high heterogeneity in our venous thromboembolism (VTE) analysis. Third, in our analysis, aspirin (ASA) dosing regimens differed across the included studies; in three studies, the aspirin (ASA) dose was 81 mg twice daily, whereas the remaining studies used 325 mg regimens. This heterogeneity in dosing regimens may influence the interpretation of our pooled results. Therefore, a subgroup analysis based on aspirin (ASA) dosing regimens was performed, in which mixed findings were observed. For deep-vein thrombosis (DVT), low-dose aspirin (ASA) was associated with higher deep-vein thrombosis (DVT) rates compared with low-molecular-weight heparin (LMWH), whereas high-dose aspirin (ASA) showed a non-significant trend toward fewer deep-vein thrombosis (DVT) events. Additionally, no significant dose-related differences were observed for pulmonary embolism (PE) or bleeding complications. However, these findings should be interpreted cautiously because the 325 mg subgroup was derived from studies with relatively small sample sizes, whereas the 81 mg subgroup was largely driven by the much larger PREVENT CLOT trial. Similarly, a previous meta-analysis demonstrated no significant difference between low-dose aspirin (ASA) and a high dose in the incidence of deep-vein thrombosis (DVT) or pulmonary embolism (PE) in elective arthroplasty procedures.

However, the incidence of overall venous thromboembolism (VTE) was significantly higher in the high-dose group than in the low-dose group [22]; this discrepancy may reflect differences in study populations and comparator settings. Existing dose-comparison meta-analyses directly compare aspirin (ASA) doses, mainly in elective arthroplasty procedures, whereas our analyses compare each aspirin dosing subgroup against low-molecular-weight heparin (LMWH) in orthopedic trauma patients. Overall, the subgroup analysis findings should be regarded as exploratory and hypothesis-generating rather than confirmatory.

Therefore, future studies should focus on conducting large-scale, multicenter randomized controlled trials with standardized dosing protocols in clearly defined orthopedic trauma populations stratified according to fracture type and location, surgical versus nonsurgical management, and baseline thromboembolic risk.

## 5. Conclusions

This meta-analysis found no significant differences between aspirin (ASA) and low-molecular-weight heparin (LMWH) in pulmonary embolism (PE), mortality, bleeding complications, wound complications, or postoperative hematoma. Aspirin (ASA) may represent a reasonable thromboprophylactic option in selected orthopedic trauma patients; however, uncertainty remains regarding overall venous thromboembolism (VTE) and deep-vein thrombosis (DVT), and the available evidence does not establish equivalent efficacy. Larger multicenter randomized trials using standardized prophylactic regimens in clearly defined and risk-stratified orthopedic trauma populations are warranted.

## Figures and Tables

**Table 1:** The summary characteristics of the included studies.

Author	Study Type	Fracture Type	Sample Size	Age (SD)	Bmi (kg/m^2^) (SD)	Intervention	Follow-Up	Outcomes
O’Toole et al. 2023 [10]	RCT	upper extremity fractures (shoulder to wrist), lower extremity fractures (hip to midfoot)	total = 12,211/ ASA = 6101/ LMWH = 6110	44.6 + 17.8/ ASA = 44.5 = 18.0/ LMWH = 44,717.6	(IQR) Total = 27.4 (23.7–32.3/ ASA = 27.1) (23.9–31.8)/ LMWH = 27.5 (23.8–32.8)	LMWH 30 mg twice daily/ ASA 81 mg twice daily	90-day follow-up	Primary outcome = any cause of death at 90 days/secondary outcome = PE, DVT, bleeding complications, wound complications, SSI
Haac et al. 2020 [1]	RCT	upper/lower extremity, pelvic, and acetabular	total = 329/ ASA = 165/ LMWH = 164	mean age = 47 ± 20	NA	LMWH = 30 mg twice daily/ ASA = 81 mg twice daily	90 days after injury	Weighted composite endpoint of death, VTE, SSI, bleeding complications
Metcalf et al. 2022 [11]	Retrospective Cohort	pelvic + acetabular	total = 144/ ASA = 54/ LMWH = 90	mean age = 42.2 ± 17.1	NA	LMWH = 40 mg twice daily/ ASA = 81 mg twice daily	Minimum 6 weeks	Primary = DVT, PE/secondary = postoperative hematoma
Jeong et al. 2007 [12]	Prospective Cohort	femoral neck + intertrochanteric	total = 917/ LMWH = 238/ ASA = 125	mean age = 79.7 ± 7.4/ ASA = 81.7 ± 7.7/ LMWH = 79.9 ± 7.7	NA	LMWH = 30 mg twice daily/ ASA = 325 mg twice daily	From surgery until hospital discharge was not mentioned	Primary = DVT, PE, fatal PE/secondary = wound infection, wound hematoma, persistent drainage, GI bleed, thrombocytopenia, mortality, intracranial bleeding
Alamiri et al. 2018 [13]	Prospective Cohort	hip fractures	total = 170/ LMWH = 54/ ASA = 116	65.61 (11.82)/ ASA 66.4 (11.52)/ LMWH 63.96 (12.37)	29.33 (10.41)/ ASA 30.7 (8.66)/ LMWH 26.24 (13.19)	ASA = 325 mg daily/ LMWH 40 mg daily	35 days post-surgery	Primary = total PE, death from PE, DVT, bleeding

Note: NA = not available.

**Table 2:** NOS of the included studies.

Study ID	Newcastle–Ottawa Scale for Cohort								
	Selection				Comparability	Outcome			Score (Out of 9)
	Representativeness of the exposed cohort (*)	Selection of the non-exposed cohort (*)	Ascertainment of exposure (*)	Demonstration that the outcome of interest was not present at the start of the study (*)	Comparability of cohorts on the basis of the design or analysis (**)	Assessment of outcome (*)	Whether follow-up was long enough for outcomes to occur (*)	Adequacy of follow-up of cohorts (*)	
Metcalf et al. 2022 [11]		*	*	*	**	*	*		7
Jeong et al. 2007 [12]	*	*		*	**	*	*	*	8
Alamiri et al. 2018 [13]	*	*		*	**	*	*	*	8

Note: * = One point awarded according to the Newcastle–Ottawa Scale, ** = up to two points awarded for comparability of cohorts.

**Table 3:** GRADE assessment of the included studies.

Outcome	Studies/N	LMWH Risk	Aspirin Risk (95% CI)	Relative Effect	Certainty	Importance	GRADE Explanation
Overall venous thromboembolism	2 studies (1 RCT, 1 cohort) N = 363	46.5/1000	24.7/1000 (1.0–374.0)	OR 0.52 (0.02–12.25)	Very low ⊕◯◯◯	Critical	Very serious inconsistency (I^2^ = 85%) and very serious imprecision due to the extremely wide confidence interval, which is compatible with substantial benefit or substantial harm. Additional risk-of-bias concerns arise from the mixed RCT and observational evidence.
Deep-vein thrombosis	4 studies (2 RCTs, 2 cohorts) N = 12,752	17.7/1000	17.7/1000 (8.2–37.4)	OR 1.00 (0.46–2.16)	Low ⊕⊕◯◯	Important	Some concerns in the RCTs and imprecision; the CI includes clinically important benefit and harm. Dose subgroup results were discordant.
Pulmonary embolism	4 studies (1 RCT, 3 cohorts) N = 12,752	15.5/1000	14.9/1000 (11.2–19.8)	OR 0.96 (0.72–1.28)	Moderate ⊕⊕⊕◯	Critical	Risk-of-bias concerns; the estimate was consistent (I^2^ = 0%) and reasonably precise around no important difference.
Fatal pulmonary embolism	3 studies (1 RCT, 2 cohorts) N = 12,608	1.3/1000	0.7/1000 (0.2–2.1)	OR 0.55 (0.18–1.65)	Very low ⊕◯◯◯	Critical	Risk of bias and very serious imprecision because only 13 total events occurred, and the CI was very wide.
All-cause mortality	2 RCTs N = 12,540	7.5/1000	7.6/1000 (5.1–11.5)	OR 1.02 (0.68–1.54)	Moderate ⊕⊕⊕◯	Critical	Some concerns in ROB2. The result was consistent (I^2^ = 0%) and dominated by the large PREVENT CLOT trial.
Bleeding complications	4 studies (2 RCTs, 2 cohorts) N = 12,937	144.8/1000	138.5/1000 (127.1–150.9)	OR 0.95 (0.86–1.05)	Moderate ⊕⊕⊕◯	Critical	Risk-of-bias concerns and variation in bleeding definitions; no statistical heterogeneity (I^2^ = 0%) and the CI was narrow.
Wound complications	3 studies (2 RCTs, 1 cohort) N = 12,903	17.5/1000	18.4/1000 (14.2–23.8)	OR 1.05 (0.81–1.37)	Moderate ⊕⊕⊕◯	Important	Risk-of-bias concerns and variable wound-outcome definitions. The estimate was consistent (I^2^ = 0%).
Postoperative hematoma	2 cohort studies N = 507	85.4/1000	32.5/1000 (12.0–86.9)	OR 0.36 (0.13–1.02)	Very low ⊕◯◯◯	Important	Observational evidence started at low certainty and was downgraded for imprecision because of the small sample and CI crossing no effect.

Certainty: ⊕⊕⊕◯ = Moderate (moderately confident); ⊕⊕◯◯ = Low (limited confidence); ⊕◯◯◯ = Very low (very little confidence).

**Table 4:** Cont. GRADE assessment of the included studies.

Outcome	Risk of Bias	Inconsistency	Indirectness	Imprecision	Publication Bias	Final Certainty	Justification
Overall venous thromboembolism	Serious	Very serious	Not serious	Very serious	Undetected	Very low ⊕◯◯◯	Downgraded for risk-of-bias concerns, very serious inconsistency (I^2^ = 85%), and very serious imprecision. The pooled estimate (OR 0.52, 95% CI 0.02–12.25) is based on only 2 studies (N = 363) and is compatible with substantial benefit or substantial harm.
Deep-vein thrombosis	Serious	Not serious	Not serious	Serious	Undetected	Low ⊕⊕◯◯	Downgraded for some concerns in the RCTs and imprecision; the CI includes clinically important benefit and harm. Dose subgroup results were discordant.
Pulmonary embolism	Serious	Not serious	Not serious	Not serious	Undetected	Moderate ⊕⊕⊕◯	Downgraded once for risk-of-bias concerns; the estimate was consistent (I^2^ = 0%) and reasonably precise around no important difference.
Fatal pulmonary embolism	Serious	Not serious	Not serious	Very serious	Undetected	Very low ⊕◯◯◯	Downgraded for risk of bias and very serious imprecision because only 13 total events occurred, and the CI was very wide.
All-cause mortality	Serious	Not serious	Not serious	Not serious	Undetected	Moderate ⊕⊕⊕◯	Downgraded for some concerns in ROB2. The result was consistent (I^2^ = 0%) and dominated by the large PREVENT CLOT trial.
Bleeding complications	Serious	Not serious	Not serious	Not serious	Undetected	Moderate ⊕⊕⊕◯	Downgraded for risk-of-bias concerns and variation in bleeding definitions; no statistical heterogeneity (I^2^ = 0%), and the CI was narrow.
Wound complications	Serious	Not serious	Not serious	Not serious	Undetected	Moderate ⊕⊕⊕◯	Downgraded for risk-of-bias concerns and variable wound-outcome definitions. The estimate was consistent (I^2^ = 0%).
Postoperative hematoma	Not serious	Not serious	Not serious	Serious	Undetected	Very low ⊕◯◯◯	Observational evidence started at low certainty and was downgraded for imprecision because of the small sample and CI crossing no effect.

Certainty: ⊕⊕⊕◯ = Moderate (moderately confident); ⊕⊕◯◯ = Low (limited confidence); ⊕◯◯◯ = Very low (very little confidence).

## Data Availability

All data are available within the manuscript and its Supplementary Materials.

## Supplementary Materials

The following supporting information can be downloaded at: https://www.mdpi.com/article/10.3390/jcm15176768/s1, Table S1: PRISMA 2020 Checklist [6].

## References

[1] Haac B.E., O’Hara N.N., Manson T.T., Slobogean G.P., Castillo R.C., O’Toole R.V., Stein D.M., ADAPT Investigators (2020). Aspirin versus low-molecular-weight heparin for venous thromboembolism prophylaxis in orthopaedic trauma patients: A patient-centered randomized controlled trial. PLoS ONE.

[2] Tarpada S.P., O’Hara N.N., Stein D.M., DeSantis A.J., Castillo R., Frey K.P., Slobogean G.P., Gitajn I.L., Gaski G.E., Kempton L.B. (2025). Effect of Aspirin Versus Low-Molecular-Weight Heparin for Thromboprophylaxis in High-Risk and Fracture Location Subpopulations: A Secondary Analysis of the PREVENT CLOT Trial. J. Orthop. Trauma.

[3] Dakay D., Gallardo E. (2023). Clinical effectiveness and safety of aspirin versus anticoagulation for venous thromboembolism prevention following orthopedic trauma and surgery: A meta-analysis of randomized controlled trials. Eur. Heart J..

[4] Haibier A., Maimaitiniyazi M., Lin H., Liu W., Li W. (2026). Aspirin vs. enoxaparin for thromboprophylaxis after total hip arthroplasty, total knee arthroplasty, or hip fracture surgery—A systematic review and meta-analysis. Front. Med..

[5] Puga T.B., Box M.W., Marchese C., Ferguson C., Poffenbarger M., Riehl J.T. (2026). Aspirin versus enoxaparin for venous thromboembolism (VTE) prophylaxis in orthopaedic trauma surgery: A systematic review. J. Orthop. Rep..

[6] Liberati A., Altman D.G., Tetzlaff J., Mulrow C.D., Gøtzsche P.C., Ioannidis J.P.A., Clarke M., Devereaux P.J., Kleijnen J., Moher D. (2009). The PRISMA Statement for Reporting Systematic Reviews and Meta-Analyses of Studies That Evaluate Health Care Interventions: Explanation and Elaboration. Ann. Intern. Med..

[7] Higgins J.P.T., Green S. (2008). Cochrane Handbook for Systematic Reviews of Interventions.

[8] Stang A. (2010). Critical evaluation of the Newcastle-Ottawa scale for the assessment of the quality of nonrandomized studies in meta-analyses. Eur. J. Epidemiol..

[9] Sterne J.A.C., Savović J., Page M.J., Elbers R.G., Blencowe N., Boutron I., Cates C.J., Cheng H.Y., Corbett M.S., Eldridge S.M. (2019). RoB 2: A revised tool for assessing risk of bias in randomised trials. BMJ.

[10] O’Toole R.V., Stein D.M., O’Hara N.N., Frey K.P., Taylor T.J., Scharfstein D.O., Carlini A.R., Sudini K., Degani Y., Major Extremity Trauma Research Consortium (METRC) (2023). Aspirin or Low-Molecular-Weight Heparin for Thromboprophylaxis after a Fracture. N. Engl. J. Med..

[11] Metcalf K.B., Du J.Y., Ochenjele G. (2022). Does Aspirin Provide Adequate Chemoprophylaxis for Venous Thromboembolic Events in Operative Pelvic and Acetabular Fractures?. Iowa Orthop. J..

[12] Jeong G.K., Gruson K.I., Egol K.A., Aharonoff G.B., Karp A., Zuckerman J.D., Koval K.J. (2007). Thromboprophylaxis after hip fracture: Evaluation of 3 pharmacologic agents. Am. J. Orthop..

[13] Alamiri M.A., Albsoul-Younes A.M., Al-Ajlouni J.M. (2018). Comparison between aspirin 325 mg and enoxaparin 40 mg as extended thromboprophylactic agents following major orthopedic surgery in Jordan University Hospital. Drugs Ther. Perspect..

[14] Stewart D.W., Freshour J. (2013). Aspirin for the Prophylaxis of Venous Thromboembolic Events in Orthopedic Surgery Patients: A Comparison of the AAOS and ACCP Guidelines with Review of the Evidence. Ann. Pharmacother..

[15] Anderson D.R., Morgano G.P., Bennett C., Dentali F., Francis C.W., García D., Kahn S.R., Rahman M., Rajasekhar A., Rogers F.B. (2019). American Society of Hematology 2019 guidelines for management of venous thromboembolism: Prevention of venous thromboembolism in surgical hospitalized patients. Blood Adv..

[16] Riediger C., Ferl M., Schönrogge M., Halm-Pozniak A. (2025). Oral Aspirin for Venous Thromboembolism Prophylaxis After Orthopedic Surgery: An Evidence-Based Review. Res. Sq..

[17] Reed C.R., Curry N., Juffermans N.P., Neal M.D. (2025). Hemostatic abnormalities after trauma resuscitation: Challenges and strategies in caring for the critically injured patient. Ann. Intensive Care.

[18] Yoshino T., Yamada Y., Cate H.T. (2023). Aspirin for Thromboprophylaxis after a Fracture. N. Engl. J. Med..

[19] Sidhu V.S., Kelly T., Pratt N., Graves S.E., Buchbinder R., Adie S., Cashman K., Ackerman I.N., Bastiras D., The CRISTAL Study Group (2023). Effect of Aspirin vs Enoxaparin on 90-Day Mortality in Patients Undergoing Hip or Knee Arthroplasty. JAMA Netw. Open.

[20] Zheng X., Li N., Song Y., Han L., Zhang Y., Yin Q., Bian Y. (2024). Comparison of efficacy and safety between aspirin and oral anticoagulants for venous thromboembolism prophylaxis after major orthopaedic surgery: A meta-analysis of randomized clinical trials. Front. Pharmacol..

[21] Schutgens R.E.G., Middeldorp S. (2023). Aspirin as Thromboprophylaxis in Orthopedic Surgery: A Matter of Perspective. HemaSphere.

[22] Mirghaderi P., Pahlevan-Fallahy M., Rahimzadeh P., Habibi M.A., Pourjoula F., Azarboo A., Moharrami A. (2024). Low-versus high-dose aspirin for venous thromboembolic prophylaxis after total joint arthroplasty: A systematic review and meta-analysis. J. Orthop. Surg. Res..

